# On the alert: future priorities for alerts in clinical decision support for computerized physician order entry identified from a European workshop

**DOI:** 10.1186/1472-6947-13-111

**Published:** 2013-10-01

**Authors:** Jamie J Coleman, Heleen van der Sijs, Walter E Haefeli, Sarah P Slight, Sarah E McDowell, Hanna M Seidling, Birgit Eiermann, Jos Aarts, Elske Ammenwerth, Robin E Ferner, Ann Slee

**Affiliations:** 1 University Hospitals Birmingham NHS Foundation Trust, Queen Elizabeth Hospital Birmingham, Mindelsohn Way, Edgbaston, Birmingham B15 2WB UK; 2 College of Medical and Dental Sciences, University of Birmingham, Birmingham B15 2SP UK; 3 West Midlands Centre for Adverse Drug Reactions, City Hospital, Dudley Road, Birmingham B18 7QH UK; 4 Erasmus University Medical Centre, Department of Hospital Pharmacy, PO Box 2040, 3000 CA Rotterdam, Netherlands; 5 Department of Clinical Pharmacology and Pharmacoepidemiology, Cooperation Unit Clinical Pharmacy, University of Heidelberg, Im Neuenheimer Feld 410, 69120 Heidelberg, Germany; 6 School of Medicine Pharmacy and Health, The University of Durham, Durham TS17 6BH UK; 7 Division of General Internal Medicine, Brigham and Women’s Hospital, Boston, MA 02120 USA; 8 Harvard Medical School, Boston, MA 02115 USA; 9 Department of Laboratory Medicine, Division of Clinical Pharmacology, Karolinska Institutet, Karolinska University Hospital, Stockholm 14186 Sweden; 10 Institute of Health Policy and Management, Erasmus University Rotterdam, PO Box 1738, 3000 DR Rotterdam, The Netherlands; 11 Institute of Health Informatics, UMIT – University of Health Sciences, Medical Informatics and Technology, Eduard Wallnöfer-Zentrum I, 6060 Hall in Tirol, Austria

**Keywords:** Clinical Decision Support Systems, Medical Order Entry Systems

## Abstract

**Background:**

Clinical decision support (CDS) for electronic prescribing systems (computerized physician order entry) should help prescribers in the safe and rational use of medicines. However, the best ways to alert users to unsafe or irrational prescribing are uncertain. Specifically, CDS systems may generate too many alerts, producing unwelcome distractions for prescribers, or too few alerts running the risk of overlooking possible harms. Obtaining the right balance of alerting to adequately improve patient safety should be a priority.

**Methods:**

A workshop funded through the European Regional Development Fund was convened by the University Hospitals Birmingham NHS Foundation Trust to assess current knowledge on alerts in CDS and to reach a consensus on a future research agenda on this topic. Leading European researchers in CDS and alerts in electronic prescribing systems were invited to the workshop.

**Results:**

We identified important knowledge gaps and suggest research priorities including (1) the need to determine the optimal sensitivity and specificity of alerts; (2) whether adaptation to the environment or characteristics of the user may improve alerts; and (3) whether modifying the timing and number of alerts will lead to improvements. We have also discussed the challenges and benefits of using naturalistic or experimental studies in the evaluation of alerts and suggested appropriate outcome measures.

**Conclusions:**

We have identified critical problems in CDS, which should help to guide priorities in research to evaluate alerts. It is hoped that this will spark the next generation of novel research from which practical steps can be taken to implement changes to CDS systems that will ultimately reduce alert fatigue and improve the design of future systems.

## Background

### Computerized physician order entry and clinical decision support

Computerized physician (or provider) order entry (CPOE) systems allow users to prescribe using a computer system, reducing the risk of prescribing errors resulting from illegible handwriting or transcription errors. They have also been shown to reduce medication errors and adverse drug reactions in hospitals [1-6], although large multi-centred trials, which give ‘guidance in optimizing CPOE implementations’ are lacking [7]. CPOE systems can have integrated clinical decision support (CDS), which attempts to improve clinicians’ decisions through guidance, alerts, and reminders. These CDS systems draw on information contained in supporting knowledge database(s), which are often integrated with software algorithms that generate alerts during drug prescribing [8,9] and may also address issues relevant for the administration process [10]. In principle, clinicians support the idea of CDS alerts in identifying and preventing erroneous or less optimal prescribing [11-14].

### Alert specificity and sensitivity

In a CDS system, sensitivity is the ability of the system to alert prescribers correctly when patients are at risk of experiencing drug-induced harm, for example, from a drug–drug interaction or drug allergy. The specificity of the CDS system is a measure of its ability to distinguish between events that put an individual at risk of harm and non-events that will not: the more false positives, the lower the specificity. Previous research has led to the suggestion that safe alerting systems should have high specificity and sensitivity, present clear information, not unnecessarily disrupt workflow, and facilitate safe and efficient handling of alerts [15]. Indeed, the ideal alert should demonstrate the following characteristics: provision of the right (correct) information, to the right person, in the right CDS intervention format, through the right channel, and at the right time in the workflow [16]. These 'five rights' may be better achieved when alerts are tailored and filtered to take into account the characteristics of (1) the organizational unit and the user, (2) the patient or case, and (3) the alert [17].

### Knowledge of alert fatigue in CDS systems

CDS alerts have the potential to cause harm to patients by occurring too frequently and thus producing distracting ‘noise’ in the system [11-14]. In most, if not all, systems a large proportion of alerts generated by CDS is overridden (i.e. clinician chooses to proceed without adjusting or cancelling the prescription) [10,15,18,19]. This may be a symptom of ‘alert fatigue’, the mental state resulting from alerts consuming too much time and mental energy, which may increase the chance that future alerts pertinent to patient safety will be overridden [20-22] along with clinically irrelevant ones [23]. In general terms, exposure to frequent false alarms can desensitize users so that they ignore and increasingly mistrust alarms [24].

Most of the focus on reducing override rates in CDS systems considers strategies such as the customization of third party providers’ sets of alerts [25-28], implementation of highly specific algorithms [18], and use of tiered severity grading to stratify and reduce the number of interruptive alerts [29,30]. Other suggested strategies to counteract alert fatigue have included turning off frequently overridden alerts and directing time-dependent drug–drug interaction alerts to nurses [31,32]. A formal framework to evaluate the appropriateness of alerts may also prove useful [33].

Alert fatigue continues to plague and frustrate users despite varying improvement strategies [34] and the overall effect and generalizability of such strategies on patient safety is unclear. Indeed, in many European countries, hospital electronic prescribing systems are in the embryonic stages, and vary widely in terms of their use and how they are being both developed and implemented. Therefore, the need for further research in the use of alerts in CDS systems remains. To address these issues, European experts on CDS attended a workshop in Birmingham, United Kingdom. Here, we describe the agreed consensus from the workshop on the current gaps in the research, the challenges of improving alerting in CDS systems, and the issues that were as yet unanswered. Recommendations are also provided for the strategic direction of future research on CDS-based safety alerts from a European perspective.

## Methods

Researchers with a strong publication record in the field of CDS were identified through literature searches and were invited by email to attend a two-day workshop in Birmingham, United Kingdom, in November 2011 on “building research capacity in the study of alerts in CDS systems”. The workshop was funded by the European Regional Development Fund and organized by the University Hospitals Birmingham NHS Foundation Trust. No ethical approval was required for this work. The objectives of the workshop were (1) to identify key knowledge gaps in the study of CDS-based alerting; (2) to identify research priorities on CDS-based alerting; and (3) to identify research methodologies to evaluate alerts.

Workshop participants (see online Additional file 1) separated into smaller groups for directed discussions after a general discussion of previous work on the nature of alert fatigue in CPOE and in other industries. Research questions to answer the objectives were discussed in each smaller participants group (N=4–6 participants) first, and then summarized in the plenum. All participants provided full consent for the recording of discussions. Minutes from the discussions were transcribed and circulated among the group for approval following the workshop. The main themes highlighted and discussed at the workshop were abstracted, and further sub-themes identified. All participants were requested to comment on various iterative drafts and their comments are incorporated into this paper. The following report reflects the discussions and recommendations of the expert participants, with additional contributions from an invitee who was unable to attend.

## Results

### Knowledge gaps in the study of alerts in CDS systems

The workshop participants identified knowledge gaps in the study of alerts in CDS systems that require further investigation (Table 1).

**Table 1:** Identified knowledge gaps in the research on CDS alerts

**Research gap**	**Comments**
1. Sensitivity and specificity of a CDS system	It is unclear whether there is an ideal sensitivity and specificity of a CDS system or whether there is an optimum number of alerts within a system.
2. Presentation and personalization of alerts	The best strategies for contextualizing, presenting and filtering alerts for users are still uncertain.
3. Timing of alerts	The appropriate point in the workflow process for alerting users needs to be determined.
4. Relevance of the outcome measures in the study of alerts	Studies on effects of alerts often include surrogate markers instead of patient parameters as outcome measures.
5. Measurement of the quality of alerts	The criteria by which the quality of an alert is judged or whether an alert adds value to a system have not been defined.
6. Design and firing of alerts/rules	A systematic approach to the generation of alerts has never been explicitly described.
7. Legal issues	The legal implications in the study of alert fatigue are yet to be established. This has been, however, discussed in an American context [35], with particular emphasis on the liability implications of CDS with drug–drug interactions [36,37].
8. Human factors and usability	More investigation of the interaction between users and CDS systems is needed.

### Important research priorities

The following four priorities for research on alerts in CDS systems have been developed from the gaps in knowledge identified in Table 1. As time was limited, the workshop did not assign research priorities to all areas where gaps in knowledge were identified.

#### Determine the optimum sensitivity and specificity of a CDS system in practice

We agreed that the perfect CDS system would be both 100% sensitive and 100% specific. Indeed clinicians will wish the overall sensitivity and specificity to be high; that is, will wish to know all those patients who will in fact experience drug-induced harm, and none of those who will suffer no harm (whether or not they are at risk). Current systems tend to have high sensitivity but often the specificity is low [38]. Sensitivities below 100% are risky and may contribute to patient harm, especially for the most injurious events. If we strive for high sensitivity, we will inevitably increase the number of alerts. Should we instead be looking for better specificity?

It is important that the system is able to draw in additional information from beyond the knowledge base – by which we mean the collection of evidence-based information about drugs and their interactions – to increase specificity, for example through the integration of individual patient information such as laboratory values and co-morbidities with information on medicines [39-42]. The challenge is in ensuring that drug information is accurate, comprehensive and up-to-date, whilst keeping the process manageable in terms of expertise, time, and resources. One solution may be the collaborative development and sharing of knowledge bases between countries [9,43]. Indeed, national knowledge bases exist, such as the Dutch national drug database (G-Standaard), which serves as a professional standard for pharmacists in the Netherlands, and is the standard from which all Dutch CPOE systems are based [44]. Sharing of such knowledge bases may enable the effective use of resources and harnessing of expertise.

However, recent findings suggest that systems can be both sensitive and specific, or indeed lack both qualities [45]. Furthermore, system quality may differ with regards to different alert categories (e.g. overdose vs. drug–drug interactions), and differences when alerting for medications only, as opposed to a combination of medication and patient parameters [45,46]. Future studies involving rigorous testing, alongside more in-depth analysis of system design features leading to high sensitivity and specificity, can be used to guide future CPOE system designs in order to determine the optimum sensitivity and specificity in the real world. By comparing differences in the design of current systems, it may be possible to identify a gold standard on which to base future CPOE systems.

Research from other industries may help guide discussions on the appropriate balance between specificity and sensitivity. A meta-analysis identified experimental studies that compared human performance aided by imperfect diagnostic automation with unaided human performance detecting the same signals — when sensitivity of automated alerts fell below 70%, performance was worse than in the absence of automation [47]. This is a rough estimate (95% confidence interval = 56–84%), but it suggests that automated systems require the sensitivity of alerts to be over some minimum value if they are to be beneficial. Indeed, this apparently counter-intuitive finding that poor CDS is worse than no CDS emphasizes the dangers of systems that miss important signals.

#### Determine whether personalization of alerts will reduce alert fatigue

Certainly, the importance of applying human factors principles to matters such as placement, visibility, prioritization, and colour in the design of CDS alerts is well established [48-50]. Customization to the setting in which the system is used (the ‘use environment’) could provide an opportunity to eliminate inappropriate alerts and requires further evaluation. For example, an interaction warning for excessive sedation by a benzodiazepine/opioid combination is rather meaningless in anaesthetized patients. Hence, such alerts would be suppressed in the “operating theatre” but not in “general practice”.

Allowing individual users to personalize the interface design of CDS alerts may also reduce alert fatigue. Indeed, CDS system developers can learn a lot from smartphones, which allow for the personalization of their user interface (e.g. alter icon arrangement, font size, or background colour). Most smartphone users enjoy the ability to modify their devices in certain ways. Conversely, we agreed that CDS alerts are often boring, difficult to see and understand, and thus frustrating to users. In addition, we discussed that CDS alerts often result in negative feeling in users, for example, because their clinical decision making has been criticized. Determining whether cosmetic personalization improves usability and receptivity of CDS alerts is important and should be investigated.

Personalization of alerts may not just be limited to the user interface, but may be done in an automatic way based upon a user’s familiarity with certain risk situations, training, and expertise. For example, frequent users may require fewer alerts than those who rarely use a system or a specific medicine. The development of individualized alerts will require structured and systematic design to ensure that they are generated appropriately for each patient. Allowing an individual prescriber to have control over which alerts are switched on or off may have some benefit, but could introduce the potential for error due to slips or lapses [51], particularly when a clinician is busy or distracted. Determining whether personalization in this way improves usability and receptivity of CDS alerts is important.

Alerts are only valuable if they may change the patient’s clinical management. Those that are irrelevant to clinical management add to the alert burden without any clinical benefit. Studies to identify and refine management decision support will be important.

#### Determine whether appropriate timing of an alert within the prescribing process will reduce alert fatigue

We agreed that ideally alerts should be displayed as early as possible in the prescribing process, and if possible, there should be no more than one alert for any prescription (by which we mean, an order for a single item). So, for example, if a situation exists that would strictly contraindicate a prescription (such as a clinically relevant allergy alert) users should be alerted before they have gone too far down the prescribing path. However, there are difficulties in a system set up to show only one alert for one drug prescription. For example, the user may have to enter all necessary information (e.g. dose, route, frequency and duration) before an overarching CDS alert relevant to all elements of the prescription is shown. Alerting the user as early as possible and having complete information that can be integrated into a single CDS alert are not easily compatible. We discussed a hierarchy of agreed alerts, that is, a grading such as (i) prescribing absolutely contraindicated; (ii) prescribe but only if certain conditions are met; and (iii) prescribe where benefit outweighs harm. Such a hierarchy would mitigate this conflict, since an alert at the highest level that interrupted the process could be displayed as soon as it was first encountered. However, this may be difficult to achieve in practice. Indeed, depending on the user interface of the computerized system, one alert per item may not be practicable. For example, when prescriptions for multiple drug items can be entered all together it could be difficult to determine which alert should be selected with priority. New research should focus on assessing the impact of the timing and number of alerts generated during one drug prescription.

#### Determine the relevance of the outcome measures in evaluating alerts

One of the main challenges in designing and evaluating alerts is deciding on what outcome measure(s) should be used. Here we suggest potential measures (Table 2).

**Table 2:** Potential outcome measures for the evaluation of alerts in CDS

**Suggested outcome measure**	**Comments**
Patient harm	This entails identifying patient harms specific to the prescribing process that may be prevented by CDS; and then establishing their relative importance.
Length of stay in hospital	This measure has the benefit of being easily measured, but depends on several factors other than the quality of prescribing.
Mortality	Again, this measure has the benefit of being easily measured, but depends on several factors other than the quality of prescribing.
Quality measures	The National Quality Forum in the USA has developed quality measurements and test cases in order to capture medical decision making and a direct link between decision process and quality of care [52].
Measures of clinical improvement	Some examples include decreased fever and falling white cell count.
Medication errors [53]	It is difficult to identify and often to define actual medication errors and perhaps even more challenging to establish the potential harm caused by these errors.
Costs	These may be an appropriate outcome measure, but the workshop’s view was that the primary aim of CDS is to minimize harm, not cost.

Although several outcome measures exist, those that are obviously relevant such as mortality, are unlikely, because of their rarity, to give a full or reliable picture of the value of alerts; and those which are more common are less obviously relevant. The correct balance needs to be established. There is also a need for consistent definitions of medication error and therapeutic harm [53] in order to increase the comparability of studies [54].

### Research methods to evaluate alerts

The appropriate research methods for evaluating alerts depend on the research question being asked. Here we consider methods that would be of potential use in evaluating alerts in CDS and for addressing the priorities for research identified during the workshop.

#### Expert opinion

Expert opinion has been previously used to try to evaluate alerts and potentially improve the quality of alerting. For example, groups of clinicians have been asked to agree on which alerts could be turned off safely within a hospital system [32] and to assess the value of alerts for 120 drug–drug interactions [55]. This method has also been used to identify and refine high-severity drug–drug interactions [56] and to identify low-priority drug–drug interactions that do not require interruptive alerts [57]. These studies may provide information on some research gaps, such as determining which outcome is most relevant to the specific research question. An expert panel could be used to examine a large number of prescriptions and see what warnings appear. The panel would select an outcome measure and explore how to reduce the number of warnings, in order to design more specific alert algorithms. A possible increase in alert adherence could then be investigated.

#### Observational (naturalistic) studies

Naturalistic studies involve the careful observation and recording of behaviours and events in their natural setting and they can be very powerful when based on strong theoretical foundations. Such studies have an important role in the study of alerts in CDS systems and previously have been used to explore factors affecting prescribing errors in hospitals [58]. A possibility for a naturalistic study would be to implement CDS systems in one geographical area or electronic health record system and study the outcome compared with other areas or groups where no system exists. Alternatively, an evaluation of the outcomes pre- and post-introduction of a CDS system in one setting may provide useful information on the effect of CDS alerts. Indeed, a recent controlled trial demonstrated a reduction in the prevalence of potentially dangerous drug–drug interactions, after implementation of a drug–drug interaction database [59]. However, a given CDS system may produce different responses in different healthcare settings [60].

#### Experimental studies

Experimental studies can allow for the manipulation and testing of CDS alerts in a controlled environment. For example, it would be possible to turn alerts on and off to see the effect on override rates. This could be undertaken in a safe setting that would not have any direct impact on patient care or safety during the experiment. A previous study found that the rates of prescribing errors fell significantly when junior doctors were shown a modal alert – an alert that requires users to interact with it before they can return to the main interface – compared with a non-modal alert [61]. There are few similar studies. They may be the most practicable way to investigate the ideal level of sensitivity and specificity, as well as determining the effects of the personalization of alerts for the user. However, they may not take into account the effect of stressful working environments on the user.

#### Comparing naturalistic and experimental study designs

Naturalistic studies have the advantage of providing a real-life assessment of alerts and alert overrides. However, removing alerts and monitoring the effect may be problematic and unethical. The effectiveness of such studies is also dependent on having a suitable audit trail and capturing the data reproducibly. Conversely, experimental studies benefit from a controlled environment with no ethical constraints. However, such studies may not reflect real-life use of alerts accurately, as prescribers could alter their use of a CPOE system when they are being watched.

#### Challenges to implementing research methods

The value of the alert must in part be judged by the actions taken in response to it. This may, however, prove challenging. There is, at present, no easy way to differentiate between an informed decision to override an alert and one that is ignored or missed, although this is a critical distinction. Some systems require a reason for overriding certain alerts – but this adds to the burden on the end user, and only provides reliable feedback to the system developers if the user provides the true reason for overriding.

Both naturalistic and experimental approaches are valuable, but the sequencing of events within the study design is important: the effectiveness of a naturalistic approach depends on having a suitable audit trail and capturing the data reproducibly. Randomization and masking in experimental studies can be difficult in such circumstances [62]. There are also problems with screen capture designs, since in naturalistic settings there are many actions to capture successfully.

It would also be beneficial to work with organizations that are looking to implement CPOE/CDS and monitor the steps to full implementation. Nonetheless, the challenge of how to measure the adverse outcomes prevented or caused by a decision support system remains, and a gold standard defined by an expert group is needed to achieve such measurement. This standard could then be used as a yardstick to judge the system. However, this may only test the system and not the outcome.

An ‘ideal’ design for studies of alerts in CDS systems may not exist. One of the approaches could be a purposeful synthesis/integration of different studies leading to new insights. However, one specific study design may provide considerable insight into CDS alerts. This would be to set up a safe environment where alerts that are always overridden are removed from a CDS system. After the alerts are removed, the system is then interrogated to see what happens in terms of override rates and potential patient outcomes. If supported by an expert panel who could assess existing alerts and select those for removal, an iterative prospective study may be valuable.

## Discussion

### Summary of findings

We have identified several research priorities including (1) the need to determine the optimal sensitivity and specificity of alerts; (2) whether adaptation to the environment or characteristics of the user may improve alerts; and (3) whether modifying the timing and number of alerts improves alerts. We have also discussed the challenges and benefits of using naturalistic or experimental studies in the evaluation of alerts and suggested appropriate outcome measures.

We recommend that the reduction of alert fatigue may be possible through the integration of patient, illness and medicine information, and through the development of an alert hierarchy to generate at most one clinically relevant alert per prescription. Ideally, alerts will only be displayed when there is a true risk of harm, but will always be displayed when such risks exist, to the extent that it is desirable or necessary for the user. Given that no practical system can achieve 100% specificity and 100% sensitivity, the best balance needs to be determined. We believe that specificity can be increased without sacrificing sensitivity through the integration and linkage of solid knowledge bases and patient parameters, using well-tailored algorithms. Future collaboration with researchers and practitioners from other countries, such as the United States, where the use of CDS systems is prominent, is also important. However, this does mean that, even if conclusions can be made about optimal alert generation, it may not be possible to implement universal change within every system in every hospital.

### Strengths and limitations of the approach

This workshop successfully facilitated collaboration and communication between researchers, which allowed for the further refinement of research priorities and the generation of future research ideas. Most discussion on CDS has come from the United States [7,43]; here we have identified and refined the knowledge gaps, particularly relevant to the European market. Workshop participants were experts in the use of CDS, with a wide breadth of knowledge of and experience in using a variety of locally-developed and commercial prescribing systems. However, as a limitation, the majority of participants were academic researchers and we did not consult other potentially relevant groups such as CDS vendors. Time constraints also meant that not every knowledge gap could be expanded upon during the workshop. Furthermore, despite a varied representation of participants from across Europe, this paper cannot, unfortunately, provide a pan-European perspective on alerts in CDS.

## Conclusions

The use of CDS systems within CPOE is increasing rapidly and is becoming an essential component of patient care in many countries. Previous research has indicated the need to eliminate alert fatigue, but this has yet to be achieved in practice. Research should be undertaken to determine whether the use of CDS alerts really improves patient outcomes, using appropriate methodologies and appropriate outcome measures. Strategies must be developed to reduce the burden of CDS alerts without compromising patient safety.

## Competing interests

All authors have no conflict of interest to declare.

## Authors’ contributions

All authors have (1) contributed to the concept of the paper; (2) drafted the paper or revised it critically for important intellectual content; and (3) have given their final approval of the submitted paper.

## Pre-publication history

The pre-publication history for this paper can be accessed here:

http://www.biomedcentral.com/1472-6947/13/111/prepub

## Supplementary Material

Additional file 1 List of Workshop Participants, affiliated institution(s) and expertise. Click here for file
