# Correction: On the alert: future priorities for alerts in clinical decision support for computerized physician order entry identified from a European workshop

**DOI:** 10.1186/1472-6947-13-122

**Published:** 2013-11-05

**Authors:** Jamie J Coleman, Heleen van der Sijs, Walter E Haefeli, Sarah P Slight, Sarah E McDowell, Hanna M Seidling, Birgit Eiermann, Jos Aarts, Elske Ammenwerth, Ann Slee, Robin E Ferner

**Affiliations:** 1 Queen Elizabeth Hospital Birmingham, University Hospitals Birmingham NHS Foundation Trust, Mindelsohn Way, Edgbaston, Birmingham B15 2WB, UK; 2 College of Medical and Dental Sciences, University of Birmingham, Birmingham B15 2SP, UK; 3 West Midlands Centre for Adverse Drug Reactions, City Hospital, Dudley Road, Birmingham B18 7QH, UK; 4 Department of Hospital Pharmacy, Erasmus University Medical Centre, PO Box 2040, 3000, Rotterdam, CA, Netherlands; 5 Department of Clinical Pharmacology and Pharmacoepidemiology, Cooperation Unit Clinical Pharmacy, University of Heidelberg, Im Neuenheimer Feld 410, 69120, Heidelberg, Germany; 6 School of Medicine Pharmacy and Health, The University of Durham, Durham TS17 6BH, UK; 7 Division of General Internal Medicine, Brigham and Women’s Hospital, Boston, MA 02120, USA; 8 Harvard Medical School, Boston, MA 02115, USA; 9 Department of Laboratory Medicine, Division of Clinical Pharmacology, Karolinska Institutet, Karolinska University Hospital, Stockholm 14186, Sweden; 10 Institute of Health Policy and Management, Erasmus University Rotterdam, PO Box 1738, 3000, DR, Rotterdam, the Netherlands; 11 Institute of Health Informatics, UMIT – University of Health Sciences, Medical Informatics and Technology, Eduard Wallnöfer-Zentrum I, 6060, Hall in Tirol, Austria

## Correction

After publication of the original article [1] it came to the publishers attention that the names of the last two authors have been inadvertently transposed. The correct authors’ list should read as indicated above. We apologize for any inconvenience this has caused.

## Pre-publication history

The pre-publication history for this paper can be accessed here:

http://www.biomedcentral.com/1472-6947/13/122/prepub
