# Supplementary material for: On the alert: future priorities for alerts in clinical decision support for computerized physician order entry identified from a European workshop
Source: BMC Med Inform Decis Mak. 2013 Oct 1;13:111. doi: 10.1186/1472-6947-13-111 (PMC3850158; doi:10.1186/1472-6947-13-111)
Supplement: Additional file 1 — List of Workshop Participants, affiliated institution(s) and expertise. [file 1472-6947-13-111-S1.docx]

Appendix - On the alert: future priorities for alerts in clinical decision support for computerized physician order entry identified from a European workshop

List of Workshop Participants, affiliated institution(s) and expertise

| Expert | Affiliated institution(s) | Expertise |
| --- | --- | --- |
| Jamie J Coleman, Consultant in Clinical Pharmacology | University Hospitals Birmingham NHS Foundation Trust; University of Birmingham; West Midlands Centre for Adverse Drug Reactions | ePrescribing; Clinical pharmacology |
| Heleen van der Sijs, Hospital Pharmacist | Erasmus University Medical Centre | CPOE; drug safety alerting; medication error; (paediatric) clinical pharmacology |
| Walter E Haefeli, Head of Department of Clinical Pharmacology & Pharmacoepidemiology | University of Heidelberg | Clinical pharmacology; individualization of drug therapy |
| Sarah P Slight, Pharmacist & NIHR Postdoctoral Research Fellow | University of Durham; Brigham and Women’s Hospital; Harvard Medical School | Clinical decision support; prescribing errors; patient safety |
| Sarah E McDowell, Research Associate | University Hospitals Birmingham NHS Foundation Trust | Medication error; epidemiology |
| Hanna M Seidling, Head of Cooperation Unit Clinical Pharmacy | University of Heidelberg | Medication errors; Prevention of overalerting |
| Birgit Eiermann, Pharmacist | Karolinska Institutet | Clinical pharmacology; knowledge databases |
| Jos Aarts, Senior Research Scientist Health Informatics | Erasmus University Rotterdam | Health informatics; impact of health information technology on clinical work practices and workflow; electronic health records; decision support systems; electronic prescribing of medications |
| Elske Ammenwerth, Professor for Health Informatics | Institute of Health Informatics, UMIT | CPOE; evaluation; IT management |
| Ann Slee, Pharmacist & Honorary Senior Clinical Lecturer | University of Birmingham | ePrescribing; medicines management; medication error |
| Robin E Ferner, Honorary Professor of Clinical Pharmacology | University of Birmingham; West Midlands Centre for Adverse Drug Reactions | Clinical pharmacology; medication error |
